# Pediatric Vernal Keratoconjunctivitis (VKC): Current State and Future Directions—A Narrative Review of Clinical Features, Diagnostic Strategies, and Emerging Therapies

**DOI:** 10.3390/children13030335

**Published:** 2026-02-26

**Authors:** Elia Pignataro, Giulia Brindisi, Alessandra Gori, Giorgio Colletti, Paola Moraca, Bianca Laura Cinicola, Alberto Spalice, Caterina Anania, Anna Maria Zicari

**Affiliations:** 1 Department of Experimental Medicine, La Sapienza University, 00161 Rome, Italy; 2 Department of Maternal Infantile and Urological Sciences, Sapienza University of Rome, 00161 Rome, Italy

**Keywords:** vernal keratoconjunctivitis, children, ocular allergy, corneal involvement, immunopathogenesis, cyclosporine A, biomarkers

## Abstract

**Highlights:**

**What are the main findings?**

- Vernal keratoconjunctivitis in childhood is a heterogeneous chronic inflammatory disease whose clinical burden and risk of corneal damage are underestimated by prevalence alone.
- Disease expression reflects the interplay between type-2 inflammation, epithelial dysfunction, and non-IgE-mediated immune mechanisms.

**What are the implications of the main findings?**

- Early recognition and severity-driven, steroid-sparing management are crucial to prevent avoidable visual morbidity in children.
- Molecular and tear-based biomarkers may enable future precision-oriented approaches to VKC diagnosis and treatment.

**Abstract:**

Vernal keratoconjunctivitis (VKC) represents far more than a typical allergic eye disease. It is a distinct and often underestimated chronic inflammatory condition that primarily affects children during critical stages of physical and emotional development. Though frequently grouped with seasonal allergic conjunctivitis, VKC differs significantly in its immunopathology, clinical presentation, and long-term implications. Its intense ocular symptoms and its potential for corneal damage and substantial psychosocial burden require, rather than symptom control, coordinated and multidisciplinary management. This narrative review explores VKC from every angle, with a particular focus on its implications for pediatric care. VKC, in fact, represents a genuine clinical challenge: as its symptoms can mimic milder forms of conjunctivitis, its course is often unpredictable, and its treatment requires balancing efficacy and safety in vulnerable age groups. We examined the immunological mechanisms that make it a model of localized Th2 inflammation, the diagnostic pitfalls that delay recognition, and the evolving treatment landscape, from conventional therapies like cyclosporine A and tacrolimus to innovative agents such as omalizumab and dupilumab. We also highlighted the role of emerging biomarkers, the influence of environmental and microbiome factors, and the urgent need for standardized care pathways. As research continues to expand our understanding, VKC is emerging as a prime example of how personalized medicine and translational science can intersect to address complex immune-mediated diseases in children. For the ones treating pediatric allergic disorders, VKC is no longer a rare curiosity: it is a clinical challenge worth understanding deeply.

## 1. Introduction

Vernal keratoconjunctivitis (VKC) is a chronic, recurrent inflammatory disease of the ocular surface that primarily affects children and adolescents, especially males, and is characterized by seasonal exacerbations and significant functional impairment [1,2]. Although traditionally considered a severe allergic conjunctivitis, VKC is now recognized as a heterogeneous condition in which both IgE-mediated and non-IgE-mediated mechanisms contribute to disease expression [3,4].

Its epidemiology varies widely, with slightly lower prevalence in temperate and colder regions and higher rates in areas with warmer and Mediterranean climates, higher ultraviolet exposure, and substantial environmental allergen burden [5,6]. Atopic comorbidities are frequent, yet a significant proportion of patients show no systemic sensitization, suggesting distinct pathogenic pathways and supporting the concept of multiple immunological endotypes [7,8].

Clinically, VKC causes intense itching, photophobia, tearing, and fluid mucus discharge, affecting daily activities and quality of life, with a risk of severe complications such as keratopathy and shield ulcers [9,10]. Advances in immunopathogenesis, including studies on the role of Th2/Th17 responses, epithelial barrier dysfunction, innate immune activation, and tear proteomics, have deepened the understanding of disease mechanisms and potentially opened new therapeutic perspectives [11,12,13].

Traditional management based on antihistamines, mast-cell stabilizers, and corticosteroids is increasingly complemented by topical immunomodulators such as cyclosporine A and tacrolimus, which help reduce steroid burden [14,15]. Novel biologics and targeted therapies are emerging but require further evidence before widespread adoption [16,17].

The aim of this narrative review is to provide an updated overview of pediatric vernal keratoconjunctivitis, integrating clinical, immunological, and therapeutic evidence. We seek to highlight its heterogeneity and to support structured severity assessment and steroid-sparing management in children. By bridging clinical practice with emerging molecular insights, this review aims to promote a more coherent and pathophysiology-driven approach to pediatric VKC, encouraging earlier recognition and more rational therapeutic decision-making.

## 2. Materials and Methods

This narrative review was based on a structured literature search conducted in the PubMed and Scopus databases. The search was performed up to December 2025 and focused on publications addressing pediatric vernal keratoconjunctivitis (VKC). The main search terms included combinations of: “vernal keratoconjunctivitis”, “pediatric”, “childhood”, “immunopathogenesis”, “corneal complications”, “cyclosporine”, “tacrolimus”, “omalizumab”, “dupilumab”, “biomarkers”, “proteomics”, and “microRNA”.

Original studies, randomized controlled trials, observational cohorts, translational research articles, consensus documents, and relevant narrative or systematic reviews were considered for inclusion. Priority was given to peer-reviewed English-language publications focusing on pediatric populations or providing clinically relevant data applicable to children.

Studies exclusively addressing adult atopic keratoconjunctivitis without relevance to VKC, non-peer-reviewed reports, and articles lacking clinical or mechanistic relevance were excluded. Case reports were included only when contributing meaningful insight into emerging therapies or rare associations.

**Figure 1:**
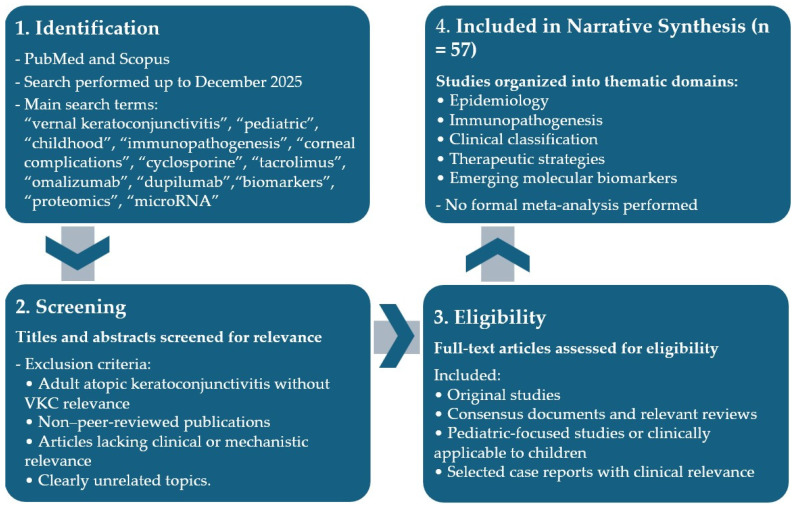
Flow diagram of literature identification and study selection process for this review.

A total of 57 publications were selected and integrated into a narrative synthesis. Given the heterogeneity of study designs, populations, and outcome measures, a formal quantitative meta-analysis was not performed. Instead, evidence was critically appraised and organized into thematic domains, including epidemiology, immunopathogenesis, clinical classification, therapeutic strategies, and emerging molecular biomarkers. The literature identification and study selection process is summarized in Figure 1.

Overall, the dataset incorporates:

- Clinical reviews offering consolidated perspectives on diagnosis, management, and disease mechanisms.
- Epidemiological and population-based studies, including data from both high-prevalence regions and areas where VKC is less frequently encountered.
- Interventional trials assessing topical immunomodulatory therapies such as cyclosporine, tacrolimus, and interferon-α2b.
- Observational cohorts and case series documenting clinical features, relapsing patterns, and corneal complications in routine practice.
- Immunological and molecular investigations examining type-2 inflammatory pathways, epithelial immune responses, and eosinophil or mast-cell activity.
- Proteomic, tear-film, and biomarker analyses, including evaluations of epithelial mediators, matrix-derived enzymes, chemokines, and microRNA signatures.
- Quality-of-life assessments, particularly those focusing on daily functioning and symptom burden in children.

## 3. Epidemiology

Vernal keratoconjunctivitis (VKC) is traditionally regarded as an uncommon form of severe allergic eye disease in temperate and high-income regions, yet studies carried out in pediatric cohorts consistently document that it represents a recognizable portion of ocular surface pathology in childhood [1,8]. European literature describes a clear male predominance, early school-age onset, and significant geographical variability, with higher frequencies in Mediterranean countries compared with northern latitudes, reflecting climatic and environmental gradients across the continent [5,18]. Importantly, even in low-prevalence settings, a meaningful share of children present with corneal epithelial changes (including punctate keratopathy), highlighting that potentially sight-threatening disease may arise independently of overall regional prevalence [5].

Outside Europe, epidemiological surveys indicate substantially greater burdens of VKC. Data from a large US analysis provide etiological clues consistent with an atopy-driven disorder. VKC occurred predominantly in boys, clustered in school-aged children, and was strongly associated with comorbid asthma, allergic rhinitis, and atopic dermatitis. Seasonal peaks in healthcare utilization further support the contribution of environmental and aeroallergen exposures to disease expression [19]. School-based cohorts from East African regions, North African and sub-Saharan contexts, where limited access to specialist care contributes to delayed diagnosis and increased risk of corneal involvement, report the same trend [10,20,21].

In Asia, VKC appears relatively frequently in tropical and subtropical climates, where intense ultraviolet radiation, consistently high temperatures, and heavy environmental allergen loads are thought to contribute to disease expression [22,23]. In fact, registry-based and environmental health studies show that fluctuations in meteorological variables and airborne pollutants correlate with seasonal peaks both in allergic and VKC conjunctivitis [6,13]. Similarly, reports from South and Southeast Asia further suggest that the combination of climate, population density, and allergen exposure shapes both prevalence and clinical severity, with a substantial proportion of children requiring long-term follow-up [24,25].

Viewed comprehensively, despite regional differences, available epidemiological data converge on several consistent pediatric features: VKC remains relatively uncommon but not exceptional in temperate–cold climates, while it is significantly more prevalent in mediterranean and hot, dry, and dusty environments; it predominantly affects boys in school age; and although frequently associated with atopy, a sizeable subset of children exhibits non-IgE-mediated disease [3,7]. Ultimately, apparent prevalence and disease severity are strongly influenced by climatic conditions, environmental exposures, and the availability of pediatric ophthalmic services, underscoring the need for regionalized approaches to early recognition and management [16,26].

## 4. Immunopathogenesis

### 4.1. Type-2 Polarization and Epithelial Alarmins

Vernal keratoconjunctivitis (VKC) is driven by a polarized type-2 inflammatory response in which epithelial cells, mast cells, eosinophils, and Th2 lymphocytes interact to sustain chronic ocular surface inflammation. Across molecular, proteomic, and cytological investigations, elevated levels of IL-4, IL-5, IL-13, and eosinophil-attracting chemokines consistently emerge as central features of the VKC inflammatory signature [4,8,12,27]. The conjunctival epithelium is not a passive target but an active orchestrator of inflammation, releasing epithelial-derived alarmins such as TSLP, IL-33, and IL-25, which skew local immunity toward Th2 predominance and amplify the responsiveness of type-2 innate lymphoid cells [11,28].

### 4.2. IgE-Dependent and Non-IgE Pathways

Mast-cell activation is an important early event, contributing to itching, hyperemia, and mucous hypersecretion; yet VKC differs from classic seasonal allergic conjunctivitis because IgE sensitization, as mentioned above, is present only in a subset of children [3,18]. Clinical cohorts and immunologic studies show that many patients experience persistent inflammation independent of allergen exposure, implying a major role for non-IgE pathways. These include sustained T-cell activation, epithelial damage, and chronic eosinophilic infiltration, which are elements that may explain the recurrent, prolonged course even in children without systemic atopy [16,29].

### 4.3. Innate Lymphoid Cells and Tissue Remodeling

Innate lymphoid cells, particularly ILC2s, have gained prominence in recent pathophysiological models. Activated by epithelial alarmins, they produce IL-5 and IL-13, supporting eosinophil survival, goblet-cell hyperreactivity, and tissue remodeling. Tear-film analyses reinforce this paradigm by demonstrating increased concentrations of eotaxin-1/2, RANTES, TARC, and MDC—chemokines that amplify recruitment and retention of Th2 cells and eosinophils [12,27]. Conjunctival and tear samples further reveal elevated MMP-9, periostin, and matrix-remodeling enzymes, reflecting epithelial barrier disruption and ongoing extracellular-matrix turnover [30,31]. Such biochemical signatures correlate with disease activity and may predict the transition from superficial punctate keratopathy to more substantive epithelial breakdown.

### 4.4. Molecular and Transcriptomic Signatures

Proteomic studies deepen what is mentioned above by revealing upregulation of pathways related to oxidative stress, innate immune activation, and metabolic reprogramming of epithelial cells. Significant alterations in tear microRNAs, implicated in epithelial integrity, cytokine signalling, and eosinophil biology, suggest broader dysregulation of epithelial–immune crosstalk [32,33]. These findings align with transcriptomic analyses showing overexpression of multiple pattern recognition receptors, including TLRs and NOD-like receptors, which heighten the ocular surface’s sensitivity to environmental triggers such as pollutants, allergens, and microbial fragments [11,13]. Such innate-immune engagement helps explain why symptoms may flare independently of allergen exposure and supports the concept of distinct VKC endotypes beyond the classical IgE-mediated model.

### 4.5. The Role of Oxidative Stress

A recent pediatric study has uncovered a new component in the immunopathogenesis of VKC: oxidative stress, which plays a role in VKC by actively supporting chronic inflammation. Researchers evaluated oxidative stress markers in pediatric patients with VKC and compared them to healthy controls. They found increased levels of oxidative stress biomarkers in children with VKC, suggesting an imbalance between oxidative and antioxidant mechanisms. These findings support the hypothesis that oxidative stress may contribute to the pathophysiology of VKC and could represent a potential target for future therapeutic strategies [34].

### 4.6. Integrated Pathophysiological Model

The convergence of epithelial activation; Th2-skewing cytokines; innate lymphoid responses; oxidative stress; and eosinophil effector mediators, including ECP and EDN, provides a coherent explanation for the relapsing nature of the disease and the heightened risk of corneal complications in susceptible children. Even in patients without detectable systemic sensitization, these interacting mechanisms generate an inflammatory micro-environment capable of sustaining chronic symptoms, disrupting epithelial repair, and predisposing to recurrent epithelial erosions and shield ulcer formation [4,12].

Overall, current evidence depicts VKC as a multifaceted inflammatory disorder in which epithelial injury, innate-immune activation, and Th2-dominated adaptive responses reinforce each other to maintain chronic inflammation. This integrated view provides the biological basis for the diverse phenotypes, variable severity, and recurrent course that characterize VKC in childhood.

## 5. Classification

The classification of vernal keratoconjunctivitis (VKC) has progressed from simple anatomical distinctions to more structured systems that incorporate inflammatory activity and the risk of corneal involvement. The classical anatomical framework, rooted in early clinical series and still widely referenced, distinguishes three principal phenotypes: (i) **tarsal VKC**, characterized by giant papillae on the upper tarsal conjunctiva; (ii) **limbar VKC**, presenting with limbal hypertrophy, gelatinous infiltrates, and Horner–Trantas dots; (iii) **mixed VKC**, in which tarsal and limbal manifestations coexist [2,35,36]. This scheme remains clinically useful because it identifies the dominant site of inflammation and reflects regional epidemiological trends, such as the higher proportion of limbal forms described in several African and Asian pediatric cohorts [20,24].

However, anatomical phenotyping alone does not adequately capture the overall severity of disease or the likelihood of sight-threatening complications, particularly in pediatric patients in whom early corneal damage may be subtle or transient. To address this limitation, severity-based classifications were introduced. One of the earliest structured grading models, proposed by Bonini and colleagues, integrated symptom intensity with objective signs, including the presence or absence of corneal involvement [36]. Within this framework, VKC may be categorized as: (i) **mild**, with intermittent symptoms, limited papillary or limbal activity, and no corneal damage; (ii) **moderate**, with more persistent symptoms, prominent conjunctival disease, and occasional superficial punctate keratopathy; (iii) **severe**, defined by frequent symptoms and definite corneal complications such as macro-erosions, shield ulcers, or fibrocellular plaques [1,10].

More recent analyses have expanded on this concept by emphasizing a three-tier severity approach that explicitly integrates activity (symptoms, hyperemia, or papillary reaction) and damage or damage risk (corneal staining, epithelial breakdown, or established keratopathy). Pediatric-focused reviews underline that these two dimensions must be interpreted together: a child with intense itching, photophobia, and marked conjunctival inflammation but an intact cornea may fall into a different severity category from one with moderate symptoms but documented corneal epithelial defects [37,38]. Contemporary management recommendations uniformly highlight that any corneal involvement in a child should be considered severe VKC, regardless of the subjective symptom burden, due to the risk of long-term consequences in visual morbidity [10,39].

Applying these classification systems in pediatric practice requires adapting to real-world clinical constraints, including limited cooperation and partial availability of slit-lamp evaluation. Practical guidance from pediatric cohorts suggests that a functional three-step assessment can be implemented by focusing on: (i) the **pattern and chronicity** of symptoms, distinguishing seasonal from perennial forms and identifying recurrent itching and light sensitivity; (ii) **visible conjunctival changes** detectable with simple lid eversion, such as papillary hypertrophy or limbal swelling; (iii) **warning signs of corneal involvement**, including reluctance to open the eyes, reports of blurred vision, pronounced photophobia, or a history consistent with previous epithelial breakdown [1,21,40].

Considering both anatomical phenotype and structured severity grading gives the opportunity of creating a shared clinical language, either for pediatricians and ophthalmologists, facilitating earlier recognition of clinically significant disease and more rational, timely therapeutic decisions in children with VKC [16,26].

## 6. Clinical Features and Complications

Vernal keratoconjunctivitis (VKC) in childhood presents with recurrent episodes of intense itching, photophobia, tearing, and mucoid discharge, typically exacerbated by outdoor exposure and warmer seasons. Itching and light sensitivity are consistently identified as the most debilitating symptoms, often interfering with reading, school participation, and sports, frequently prompting early medical attention [1,8,41]. Children may also report burning, foreign-body sensation, eyelid heaviness, and symptoms upon awakening due to overnight accumulation of inflammatory secretions [26,39].

Ophthalmic examination reveals a recognizable constellation of signs. In the tarsal form, giant papillae on the upper tarsal conjunctiva are the defining feature, whereas limbal hypertrophy, gelatinous limbal infiltrates, and Horner–Trantas dots typify the limbal variant [24,35,36]. Mixed forms demonstrate both patterns and may shift over time depending on activity. Conjunctival hyperemia, chemosis, and ropy discharge are common during flares. Assessment in younger children can be challenging because of photophobia or limited cooperation, and repeated examinations are often required to accurately quantify disease severity [37,40].

Corneal involvement represents the most clinically significant component of VKC. A continuum of findings has been described, ranging from superficial punctate epithelial erosions to macro-epithelial defects, shield ulcers, and, in more advanced cases, fibrocellular plaques encroaching on the visual axis [1,10,30]. Shield ulcers typically arise from frictional microtrauma induced by giant papillae in combination with persistent eosinophilic inflammation. Children with severe corneal disease often exhibit marked photophobia, maintain a head-down posture, or keep their eyes partially closed; many require sunglasses indoors to mitigate light sensitivity [10,23].

Repeated epithelial injury may lead to chronic or structural sequelae. Longitudinal pediatric observations report persistent epithelial defects, irregular astigmatism, and occasionally permanent corneal scarring, particularly in cases with delayed diagnosis or insufficient control of inflammation [10,25]. The therapeutic use of topical corticosteroids, frequently unavoidable for acute exacerbations, introduces additional risks, including steroid-induced ocular hypertension, early posterior subcapsular lens changes, and increased intraocular pressure, although such complications remain relatively uncommon when treatment is carefully monitored [14,39].

Comorbid conditions commonly accompany VKC. Pediatric cohorts document elevated rates of asthma, allergic rhinitis, food allergy, and atopic dermatitis, suggesting that VKC frequently arises within a broader atopic framework [7,21,42]. A subset of children also shows non-IgE-mediated inflammatory patterns consistent with non-allergic airway disease [43]. These comorbidities may intensify symptom burden and predispose children to recurrent flares. The overall quality-of-life impact is substantial: children often experience sleep disruption, activity limitation, emotional distress, and school absenteeism during periods of heightened inflammation [9,40,41].

Altogether, the clinical profile of VKC combines recurrent symptoms, distinctive conjunctival findings, and a significant risk of corneal involvement. Early identification of red flags such as severe photophobia, refusal to open the eyes, or visible corneal opacity is essential for preventing progression to sight-threatening complications and ensuring timely referral to the ophthalmologist.

## 7. Diagnostic Approaches

The diagnosis of vernal keratoconjunctivitis (VKC) relies on a structured combination of clinical history, symptom pattern, and targeted ocular examination, incorporating diagnostic elements repeatedly described across pediatric cohorts and observational studies [8,26,35,37].

A careful symptom-oriented history supports early recognition of VKC even in children who present repeatedly with ocular itching or photophobia despite prior use of antihistamines. Observational works highlight that intensity and chronicity, rather than seasonality alone, help distinguish VKC from milder allergic conjunctivitis [7,26]. As mentioned above, on examination, diagnostic criteria consistently reported across classical and contemporary pediatric series include giant upper-tarsal papillae, limbal hypertrophy, Horner–Trantas dots, and varying degrees of superficial keratopathy—features forming the cornerstone of VKC diagnosis [24,35,36]. In addition, across clinical studies, disease severity has been assessed through composite symptom-sign scoring systems integrating itching, photophobia, conjunctival hyperemia, papillary reaction, and corneal staining, with corneal involvement consistently serving as a key marker of therapeutic escalation and overall disease burden [8,36,41].

Because cooperation may be limited in younger children, several studies emphasize the importance of repeated examinations, strategic reduction in ambient light, and systematic eyelid eversion to avoid missing subtle epithelial defects or early plaque formation [37,40]. Corneal evaluation is essential: punctate keratopathy, macro-erosions, or early shield ulcer development often appear only during flares, necessitating follow-up visits timed to symptom recurrence [10,30]. Functional assessment, including visual acuity, contributes to early detection of amblyogenic risk in cases of persistent keratopathy [39].

The association between VKC and atopic conditions has diagnostic relevance. Multiple cohorts document elevated rates of allergic rhinitis, asthma, atopic dermatitis, and other allergic diseases among affected children [7,21]. Reported sensitization to aeroallergens varies across regions, but atopy remains consistently more frequent in VKC than in the general pediatric population, influencing both disease chronicity and the threshold for specialist referral [3,8]. In addition to clinical symptom-sign integration, emerging studies have explored tear-based molecular markers—including MMP-9, periostin, and selected microRNAs—as potential objective correlates of disease activity, although their role remains confined to research settings and has not yet entered routine clinical practice [12,31,32].

### 7.1. Laboratory and Ancillary Testing

Although VKC remains a fundamentally clinical diagnosis, ancillary diagnostic investigations described across pediatric studies can provide supportive information in selected cases. Cytological analysis of conjunctival scrapings has repeatedly demonstrated elevated eosinophils, mast cells, and inflammatory epithelial changes during active disease, offering a useful adjunct in atypical presentations or when differentiating VKC from chronic infectious or irritative conjunctivitis [29,44].

Tear-film biomarkers have gained attention as research tools capable of capturing inflammatory activity. Numerous studies document elevated levels of MMP-9; eotaxin-1/2; periostin; and a variety of chemokines, including TARC, MDC, and RANTES, markers reflective of epithelial stress and type-2 inflammatory responses [12,27,31]. Alterations in tear microRNA expression, particularly in pathways involving epithelial integrity and eosinophil signalling, have also been reported and may serve future roles in severity stratification or monitoring therapeutic response [32,33].

Advanced molecular techniques such as proteomic profiling and multiplex cytokine analyses have been applied in small cohorts, revealing reproducible upregulation of Th2 cytokines (IL-4, IL-5, IL-13), increased eosinophil-attracting chemokines, and heightened expression of alarmins and innate-immune receptors, consistent with transcriptomic findings demonstrating overexpression of multiple pattern-recognition pathways in VKC [11,13]. Although not routinely employed in clinical practice, these approaches deepen understanding of disease mechanisms and may inform future diagnostic algorithms, especially in the context of emerging targeted therapies.

Quality-of-life and symptom-severity questionnaires validated in pediatric settings complement clinical and molecular assessment. Instruments tailored to children and caregivers quantify functional limitations, emotional distress, school absenteeism, and sleep disturbance, providing an additional dimension to diagnostic evaluation [9,41].

### 7.2. Differential Diagnosis

The differential diagnosis of VKC includes several pediatric ocular surface disorders with overlapping features. Chronic allergic conjunctivitis, atopic keratoconjunctivitis, and seasonal/perennial allergic conjunctivitis can mimic early VKC but typically lack the combination of giant tarsal papillae, limbal infiltrates, and the significant risk of corneal ulceration that characterizes moderate-to-severe VKC [35,36]. Infectious keratoconjunctivitis—particularly adenoviral disease—may present with photophobia and punctate keratopathy but is distinguished by its acute onset, systemic symptoms, and characteristic conjunctival distribution [7,26].

Other pediatric entities described in diagnostic reviews include blepharokeratoconjunctivitis, toxic or mechanical keratopathy, and ocular rosacea, each requiring distinct therapeutic strategies [37,40]. Recognizing the clinical hallmarks of VKC, such as Trantas dots, prominent giant papillae, chronic relapsing symptoms across multiple seasons, and limited response to standard anti-allergic therapy, is essential to avoid misclassification and ensure timely referral for specialized care [1,8].

## 8. Therapeutic Management

The therapeutic management of vernal keratoconjunctivitis (VKC) in childhood is organized along a structured, stepwise pathway in which treatment intensity is adjusted according to disease activity and the risk of corneal involvement. Recent pediatric-focused reviews emphasize that effective management should combine early identification of the disease, rapid symptom control along with ocular surface protection, and long-term safety, with a strong emphasis on minimizing cumulative steroid exposure in children who may require therapy across several years [26,38,39].

### 8.1. Topical H1-Antihistamines

Topical H1-antihistamines are typically employed as the first pharmacologic option in children with mild VKC or early seasonal exacerbations. These agents provide rapid yet partial symptomatic relief, particularly for itching and conjunctival redness, and are generally well tolerated in pediatric practice [1,26]. Although controlled data in VKC remain limited, studies centring on allergic conjunctivitis and mixed-population cohorts support their role in partially reducing early inflammatory symptoms and improving short-term comfort.

Newer antihistamines, including agents with combined antihistaminic and mast-cell-stabilizing properties, have shown favourable safety profiles and rapid onset of action, making them reasonable options in children with intermittent symptoms or as adjunctive therapy in more complex cases [35]. In routine pediatric care, topical antihistamines can be used as monotherapy in mild disease or combined with mast-cell stabilizers during high-exposure periods to improve symptom control and reduce reliance on corticosteroids [8,36].

### 8.2. Mast-Cell Stabilizers

Mast-cell stabilizers such as cromolyn sodium, nedocromil, and lodoxamide play a preventive role by reducing mast-cell degranulation and the downstream release of histamine, leukotrienes, and prostaglandins. Cytological and biochemical studies indicate that these agents are associated with reductions in eosinophil activation, mast-cell mediators, and tear inflammatory markers during active disease [12,44].

Clinical series in high-prevalence regions describe improvement in itching, photophobia and hyperemia with lodoxamide and cromolyn, with good tolerability over repeated seasonal courses [24,40]. Although slower than antihistamines in producing symptom relief, mast-cell stabilizers are favoured for long-term maintenance, especially in children with predictable seasonal flares or recurrent episodes. They are frequently combined with H1-antihistamines early in the disease to limit subsequent steroid exposure [1,36].

### 8.3. Topical Corticosteroids

Topical corticosteroids remain the most powerful agents for the rapid control of acute inflammatory exacerbations, particularly when corneal involvement or severe photophobia is present. Their potent anti-inflammatory effects can rapidly reverse conjunctival and tarsal inflammation, often within days of initiation [10,30]. However, their use in pediatric VKC is limited by concerns regarding steroid-induced ocular hypertension, early lens opacities, and increased susceptibility to infection. Pediatric cohorts have documented a non-negligible rate of ocular hypertension even after relatively short steroid courses, underscoring the need for regular tonometry and cautious tapering [14,39]. In selected severe tarsal disease, supratarsal corticosteroid injections (typically triamcinolone) have been used with sustained reductions in giant papillae and improved comfort, though this approach requires expert supervision and stringent follow-up [2,40]. Contemporary management strategies therefore recommend short, intensive “pulse” therapy during acute flares, followed by rapid transition to immunomodulatory agents such as cyclosporine A or tacrolimus [26,38].

### 8.4. Topical Cyclosporine A (CsA)

Topical cyclosporine A is the preferred steroid-sparing therapy for moderate-to-severe VKC in childhood. It has been evaluated in multiple formulations, ranging from 0.05% to 2%, including oil-based vehicles and cationic emulsions. Studies using 1–2% CsA in pediatric VKC have reported marked improvement in composite symptom scores, reductions in tarsal and limbal inflammation, and substantial decreases in steroid requirements, with many children discontinuing corticosteroids entirely after CsA introduction [8,15,45]. A randomized controlled trial in pediatric patients demonstrated the superiority of CsA over standard therapy in reducing seasonal flares and improving corneal integrity, with a favourable safety profile [45].

Ethanol-free cyclosporine galenic eye drops exhibit a significantly improved stability, sterility and expected tolerability compared with traditional hydroalcoholic formulations, supporting their use in pediatric VKC when commercial products are unavailable, less effective or poorly tolerated [46]. More recently, a multicentre pediatric trial evaluating 0.1% CsA cationic emulsion (VEKTIS) showed clinically relevant improvement in both objective signs and symptoms, along with reduced need for rescue steroids; pooled analyses reinforced these findings across different populations [47]. Evidence supporting topical cyclosporine A in pediatric VKC is primarily derived from randomized controlled trials and pooled analyses, providing a relatively robust level of clinical evidence compared to other steroid-sparing agents.

### 8.5. Topical Tacrolimus

Tacrolimus, a more potent calcineurin inhibitor than CsA, is available as eyelid ointment or topical eye drops. It is particularly useful in children with refractory VKC or predominant tarsal disease, as prospective pediatric series have shown that tacrolimus ointment reduces itching, redness, and papillary hypertrophy with sustained responses across months, though transient burning or irritation is common initially [46,48,49]. Tacrolimus is generally reserved for cases unresponsive to CsA, or for children requiring more potent immunomodulation, and its initiation should occur under specialist supervision: randomized controlled trials evaluating tacrolimus eye drops have demonstrated significant improvements in symptom scores, inflammatory signs, and decreased steroid dependence compared with control regimens [49,50]. In contrast to cyclosporine, much of the evidence for topical tacrolimus is based on smaller prospective studies, retrospective series, and real-world experiences, with limited large-scale randomized data in pediatric populations.

### 8.6. Interferon-α2b (Topical)

Interferon-α2b eye drops have been explored as adjunctive “rescue” therapy in small pediatric cohorts with severe, refractory VKC (particularly in those with shield ulcers or persistent epithelial defects) despite maximal treatment. Reports show progressive improvement in conjunctival inflammation and enhanced corneal epithelial healing when interferon-α2b is added to background therapy, allowing for gradual steroid tapering [23,24]. However, current evidence for interferon-α2b is largely limited to small case series and pilot studies, and its use remains confined to highly selected cases managed in specialized centres.

### 8.7. Anti-IgE Therapy (Omalizumab)

Omalizumab has gained attention due to the involvement of IgE-mediated mechanisms in a subset of VKC patients, especially those with comorbid asthma, allergic rhinitis, or atopic dermatitis: pediatric series have documented marked improvement in itching, photophobia, conjunctival hyperemia, and papillary inflammation among children receiving omalizumab for systemic allergic diseases, with a significant reduction in topical steroid use [16,51]. Even though data on anti-IgE therapy in VKC derive predominantly from case reports and small uncontrolled cohorts, the consistency of benefit suggests that omalizumab may be considered in selected children whose systemic atopic disease already justifies anti-IgE therapy.

### 8.8. IL-4/IL-13 Pathway Blockade (Dupilumab)

Dupilumab, which blocks IL-4 and IL-13 signalling, has revolutionized treatment for atopic dermatitis, but its effects on the ocular surface are complex. Reports describe both marked improvement of VKC-like inflammation and, conversely, the emergence of dupilumab-associated conjunctivitis and blepharitis [16,52]. Ideally, in severe atopic dermatitis and vernal-like keratoconjunctivitis, partial or complete resolution of ocular symptoms after initiating dupilumab might be possible, suggesting potential benefit in selected cases; however, McCann et al. reported worsening ocular surface inflammation, a generally known possible side-effect nowadays, indicating the need for careful patient selection [17,53]. At present, dupilumab should not be considered standard VKC therapy and should be reserved for children with severe atopic dermatitis whose ocular disease remains burdensome despite optimal topical therapy. In fact, evidence regarding IL-4/IL-13 pathway blockade in VKC remains limited and partly extrapolated from atopic dermatitis cohorts, with heterogeneous ocular outcomes reported in the literature.

### 8.9. Putting It Together: A Stepwise Therapeutic Pathway

Considered the above mentioned data, it seems clear that the therapy should be elected according gravity, relying on a patient-centred approach, by making both children and parents understand the rationale under the election of a certain therapy [54]: In mild VKC (itching, mild redness, no photophobia, and no keratopathy), first-line treatment includes topical antihistamines alone or combined with mast-cell stabilizers, typically sufficient during seasonal flares, even though cyclosporine A and brief steroid pulses should be administered as well. In moderate VKC (persistent symptoms and tarsal or limbal inflammation without corneal involvement), escalation to topical immunomodulation, primarily cyclosporine A with brief steroid pulses for acute worsening, is paramount. In severe or refractory VKC (any corneal involvement or disabling photophobia), urgent initiation of short steroid pulses plus intensive CsA or tacrolimus, and prompt ophthalmology referral is the most advisable solution. A severity-based clinical framework integrating disease classification, key clinical features, and therapeutic measures in pediatric VKC is summarized in Table 1.

## 9. Future Directions: Molecular and Proteomic Biomarkers

Several tear-film and proteomic studies have identified emerging molecular signatures that may enhance the assessment of VKC in children. Tear analyses consistently demonstrate elevated MMP-9, eotaxin-1/2, and periostin during active disease, with strong correlations to inflammatory burden and early corneal involvement [12,30]. Proteomic investigations, including quantitative platforms and high-resolution mass spectrometry, have revealed broader alterations involving epithelial-stress pathways, extracellular-matrix remodeling proteins, and oxidative-stress markers, suggesting that multi-analyte panels may provide superior discrimination compared to single biomarkers [22,28].

Beyond chemokines and metalloproteinases, tear proteomic profiling has shown increased levels of eosinophil-derived proteins (ECP, EDN), S100 family proteins, and matrix components such as fibronectin and laminin fragments, which appear to track with epithelial vulnerability and risk of shield ulcer formation [12,27]. Additional markers related to epithelial barrier integrity, including altered mucins and junctional proteins, have been noted in proteomics-based analyses of allergic ocular disease and may hold relevance for VKC endotyping [33].

Moreover, recent multi-omics work on ocular surface inflammation, although conducted in infectious keratitis, has shown how integrated transcriptomic and tear cytokine profiling can uncover highly specific inflammatory signatures, including marked upregulation of CXCL8/IL-8, IL-1B, CXCR2, complement-related genes and IL-17-linked pathways [55]. These methodological approaches, already capable of discriminating disease activity and severity in other corneal disorders, are likely applicable to VKC and support the development of multi-analyte biomarker panels able to capture both epithelial stress and immune-regulatory dysfunction.

More recent work on tear microRNA signatures has identified numerous dysregulated miRNAs linked to pathways governing epithelial stability, Th2 polarization, and eosinophil recruitment, several of which demonstrate promising diagnostic performance and potential utility for anticipating disease reactivation [32,33,56]. These findings align with broader evidence that shows how specific tear microRNAs can regulate key immune pathways, particularly mast-cell activation, eosinophilic recruitment, and Th2-mediated inflammation. A recent review also highlighted that multi-miRNA panels may discriminate more accurately between active and quiescent inflammatory states compared with single biomarkers, supporting the development of integrated diagnostic models based on tear miRNA profiling [57]. However, although these molecular approaches are promising, most candidate biomarkers lack standardization, external validation, and cost-effectiveness data, and therefore remain largely restricted to research settings rather than routine pediatric practice. Nevertheless, the mentioned points point toward future diagnostic frameworks that integrate clinical scoring with molecular indicators of inflammatory activity, epithelial stress, and damage risk. This offers a pathway toward personalized diagnostic and therapeutic strategies in pediatric VKC. A detailed overview of emerging tear-based and molecular biomarkers is provided in Table 2.

## 10. Discussion

Vernal keratoconjunctivitis in childhood emerges as a far more complex and heterogeneous disorder than the traditional label suggests [2,4]. Across epidemiological, immunological, and clinical evidence, a coherent picture takes shape: VKC should be regarded as a chronic inflammatory disorder that requires early recognition, structured severity assessment, and timely introduction of steroid-sparing therapies [1,10]. Despite its relatively low prevalence in some regions, VKC imposes a disproportionate burden on children and families, often interfering with school, recreation, and daily function [9,41]. This discrepancy between prevalence and impact underscores one of the recurring themes of the literature: VKC remains widely under-recognized and frequently underestimated outside specialist settings [8,39].

Diagnostic challenges persist, particularly in younger patients, where intense photophobia, eyelid squeezing, and limited cooperation hinder adequate ocular examination [1,40]. The disorder’s overlapping features with more benign forms of allergic conjunctivitis often delay appropriate diagnosis, contributing to a possibly avoidable corneal morbidity [36,47]. Several clinical series emphasize that subtle signs of epithelial involvement may be missed without repeated assessments and careful lid eversion, highlighting the importance of early referral pathways and shared management between specialists [10,46]. The chronic relapsing pattern, observed in many children, further complicates the clinical picture, as symptom variability can obscure disease severity and mask progressive corneal changes [16,26].

Insights from immunopathogenesis help explain this clinical heterogeneity. The prominence of type-2 inflammation, involving Th2 lymphocytes, mast cells, eosinophils, and epithelial-derived alarmins, shapes a milieu in which episodic flares can occur in both IgE-sensitized and non-sensitized children [4]. In fact, the evidence that many patients lack detectable IgE sensitization reinforces the concept that VKC extends beyond classical allergen-driven pathways, representing a multidimensional inflammatory disease (rather than a unique entity) involving innate immune responses, epithelial dysfunction, and dysregulated tissue repair [7,47]. This view aligns well with molecular data showing altered expression of inflammatory mediators, pattern-recognition receptors, and tear biomarkers in active disease [11,12,27,33].

Therapeutic strategies have evolved in parallel with these insights. Although topical antihistamines and mast-cell stabilizers remain useful for mild disease, immunomodulatory therapy now represents the cornerstone of management in moderate-to-severe VKC [8,40]. Cyclosporine A, particularly in optimized formulations designed for ocular surface delivery, consistently reduces inflammatory activity and decreases reliance on corticosteroids [14,45,47]. Tacrolimus offers additional benefit in refractory tarsal disease [48,49,50]. while corticosteroids maintain a role as short, targeted rescue therapy rather than long-term treatment [10,39]. Interferon-α2b and systemic biologics appear promising only in highly selected cases and underscore the need for individualized approaches in children with complex disease or significant comorbid atopy [24,51,53]. However, despite these literature-based indications, real-world practice often remains fragmented, with wide variability in therapeutic choices and inconsistent monitoring, especially regarding the early detection of steroid-induced complications [36,39].

A particularly compelling frontier lies in the development of biomarkers capable of refining diagnosis and guiding treatment decisions. Tear proteomics, transcriptomics, and microRNA profiling have revealed distinct molecular signatures associated with disease activity, epithelial stress, and risk of reactivation [12,28,32,57]. However, while emerging molecular insights may refine future risk stratification, current management must prioritize structured severity grading and individualized treatment strategies in daily pediatric practice [27,33].

Altogether, the evidence synthesized in this review suggests that VKC in childhood should be reframed as a chronic, multifaceted inflammatory disorder requiring early recognition, structured severity assessment and, ultimately, personalized therapeutic strategies.

## 11. Limitations of This Review

This review has several limitations that should be acknowledged. First, its narrative design does not follow a formal systematic review framework, and therefore, study selection was not performed according to predefined PRISMA criteria. Although a structured PubMed search strategy was applied, the inclusion process inherently reflects a degree of interpretative selection.

Second, the literature on pediatric VKC is characterized by substantial heterogeneity in study design, population characteristics, severity grading systems, and outcome measures. Randomized controlled trials coexist with small observational cohorts, retrospective analyses, and case reports, limiting the possibility of uniform comparison across therapeutic strategies.

Furthermore, emerging areas such as tear proteomics, transcriptomics, and microRNA profiling remain largely confined to translational or exploratory research settings, often involving limited sample sizes. As a result, conclusions regarding biomarker applicability in routine clinical practice must be interpreted cautiously.

Finally, real-world management variability across different healthcare systems may not be fully captured by published data, particularly regarding long-term monitoring and steroid-related complications.

Despite these limitations, the synthesis presented here integrates the current immunological, clinical, and therapeutic evidence in a clinically oriented framework relevant to pediatric practice.

## 12. Conclusions

VKC reveals itself as a structurally complex disease, which challenges the boundaries between allergy and chronic ocular surface inflammation. The synthesis of current evidence suggests that our traditional, symptom-driven models of diagnosis and management are reaching their limit.

In fact, as molecular data continue to expand, pediatric vernal keratoconjunctivitis is increasingly being interpreted not only through clinical phenotypes but also through measurable inflammatory profiles, even if largely confined to research settings. Emerging evidence suggests that the challenge ahead lies in integrating such elements: tear proteomics, transcriptomics, and microRNA profiling may, in the future, contribute to earlier identification of children at risk of severe disease or corneal involvement, leading to more targeted, steroid-sparing, tailored therapies.

## Figures and Tables

**Table 1:** Clinical severity stratification and management of pediatric vernal keratoconjunctivitis.

Clinical Domain	Clinical Classification	Key Clinical Features	Therapeutic Measures
**Mild disease**	Mild VKC (no corneal involvement)	Intermittent itching and redness; mild conjunctival hyperemia; absence of disabling photophobia; no epithelial defects or shield ulceration [1,8,24,29]	Topical antihistamines ± mast-cell stabilizers for symptomatic control; seasonal/intermittent regimens when appropriate [3,8,20,34]
**Moderate disease**	Moderate VKC (persistent inflammation ± superficial keratopathy)	Persistent/recurrent itching and photophobia; evident tarsal and/or limbal inflammation; superficial punctate keratopathy may be present, but without macro-erosions or shield ulcers [8,10,24,25]	Introduce steroid-sparing immunomodulation (topical cyclosporine A as maintenance); short topical corticosteroid “pulses” for flares with close monitoring [14,15,20,23,45,47]
**Severe disease**	Severe VKC (any clinically relevant corneal involvement)	Marked photophobia and functional limitation; corneal epithelial damage (macro-erosions), early shield ulceration or plaques; risk of visual impairment and long-term sequelae [10,31,36]	Intensive anti-inflammatory strategy: short, supervised steroid pulses plus immunomodulators (cyclosporine A or tacrolimus) to reduce steroid dependence; prompt ophthalmology follow-up for corneal disease and complications [10,14,45,48,49]
**Refractory disease**	Severe, treatment-resistant VKC	Persistent inflammation and/or recurrent corneal involvement despite optimized topical therapy; frequent relapses requiring repeated rescue treatments; often within a broader atopic context in selected patients [16,25,37]	Escalation in specialist centres: optimize/step-up immunomodulators (CsA formulations or tacrolimus); consider interferon-α2b in selected severe cases; evaluate systemic options (e.g., anti-IgE) only in highly selected patients with significant comorbid atopy [16,35,50,51,52,53]

**Table 2:** Emerging tear-based and molecular biomarkers investigated in pediatric vernal keratoconjunctivitis.

Clinical Domain	Biological Matrix	Key Biomarkers	Pathophysiological Meaning	Potential Clinical Role
**Tear proteomics**	Tear fluid; proteomic profiling	Differential protein signatures distinguishing active and quiescent disease states [12,39]	Reflects inflammatory burden, epithelial stress, and risk of disease reactivation [12]	Activity monitoring and relapse-risk stratification beyond clinical scores [12,39]
**Conjunctival transcriptomics**	Conjunctival tissue; transcriptome analysis	Overexpression of pattern-recognition receptor and innate immune pathways [11]	Captures sustained innate immune activation contributing to chronicity and heterogeneity [11]	Mechanistic endotyping and identification of novel therapeutic targets [11]
**Tear microRNAs**	Tear fluid; microRNA profiling	Dysregulated multi-miRNA profiles associated with VKC activity [27,56]	Post-transcriptional regulation of epithelial–immune crosstalk in type-2 inflammation [27]	Development of multi-miRNA panels for activity assessment and monitoring [27,56]
**Tear cytokines/immune mediators**	Tear fluid; multiplex immune assays	Composite cytokine and mediator patterns rather than single markers [38,41,54]	Mirrors local ocular surface immune milieu and inflammatory activation [38]	Complementary objective measure to support severity grading in research settings [38,41]
**Tissue remodeling and injury markers**	Tears and ocular surface assays	MMP-related activity and remodeling-associated signals [12,21,41]	Indicates epithelial damage and corneal stress linked to severe disease [21]	Damage risk assessment integrated with inflammatory activity markers [12,21]
**Type-2-associated tissue signals**	Ocular surface context	Periostin as a marker of type-2 inflammation and remodeling [28,41]	Links chronic inflammation with structural tissue changes [28]	Candidate component of future composite biomarker panels [28,41]
**Ocular surface cytology**	Conjunctival scraping; cytology	Eosinophils and inflammatory cellular patterns [44]	Direct cellular correlate of local type-2 inflammation [44]	Adjunctive tool for phenotype confirmation and follow-up in specialist care [44]

## Data Availability

No new data were generated.
